# The Brief Case: Postoperative pulmonary infection caused by *Bordetella hinzii*

**DOI:** 10.1128/jcm.00695-25

**Published:** 2025-11-12

**Authors:** Jie Zhou, Huaming Peng, Lixian Ye, Baohu Zhang, Yang Zhang, Li Zhang, Yi Qian, Shucai Yang

**Affiliations:** 1 Department of Clinical Laboratory, Pingshan Hospital, Southern Medical University (People’s Hospital of Pingshan Shenzhen) 647773 , Shenzhen, Guangdong, P.R. China; 2 Intensive Care Unit, Pingshan Hospital, Southern Medical University (People’s Hospital of Pingshan Shenzhen) 647773 , Shenzhen, Guangdong, P.R. China; 3 Department of General Practice, Pingshan Hospital, Southern Medical University (People’s Hospital of Pingshan Shenzhen) 647773 , Shenzhen, Guangdong, P.R. China; Endeavor Health, Evanston, Illinois, USA

**Keywords:** *Bordetella hinzii*, pulmonary infection, antimicrobial susceptibility test

## CASE

A 50-year-old man was admitted to the Department of Cardiothoracic Surgery following trauma sustained in a fall from a significant height. He sustained multiple right-sided rib fractures, bilateral pulmonary contusions, and right-sided pneumothorax with associated hemothorax. The patient underwent video-assisted thoracoscopic surgery (VATS) for right lung wedge resection, along with open reduction and internal fixation (ORIF) of the rib fractures under general anesthesia. The surgical procedure was uneventful, and the patient was subsequently transferred to the intensive care unit (ICU) for postoperative monitoring and ventilatory support via endotracheal tube.

**Fig 1:**
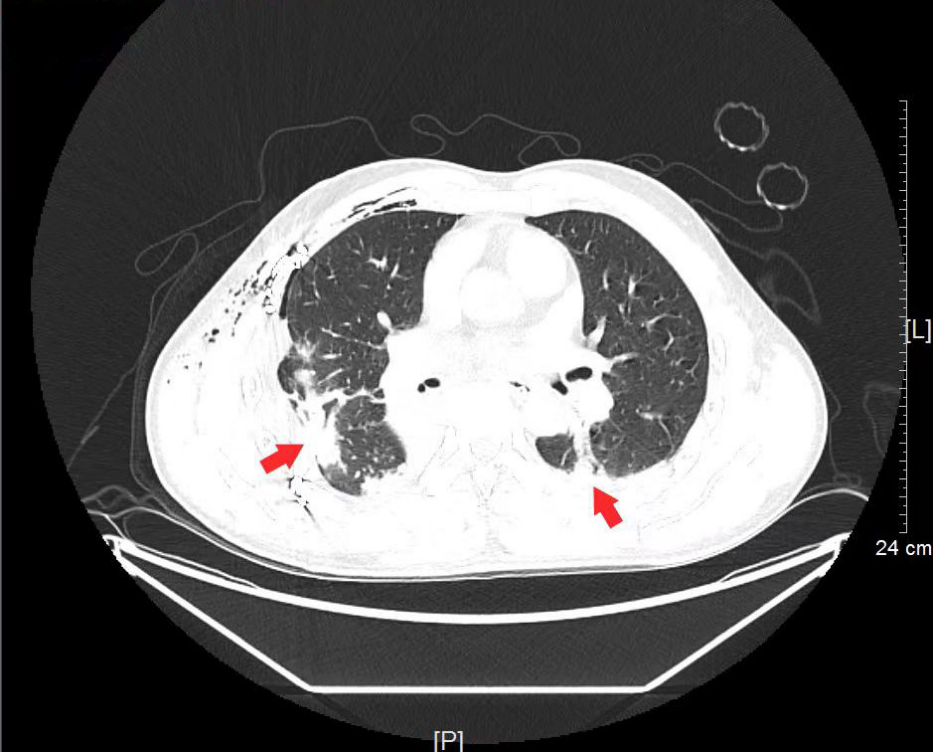
Chest computed tomography (CT) scan of the patient infected with *B. hinzii*.

**Fig 2:**
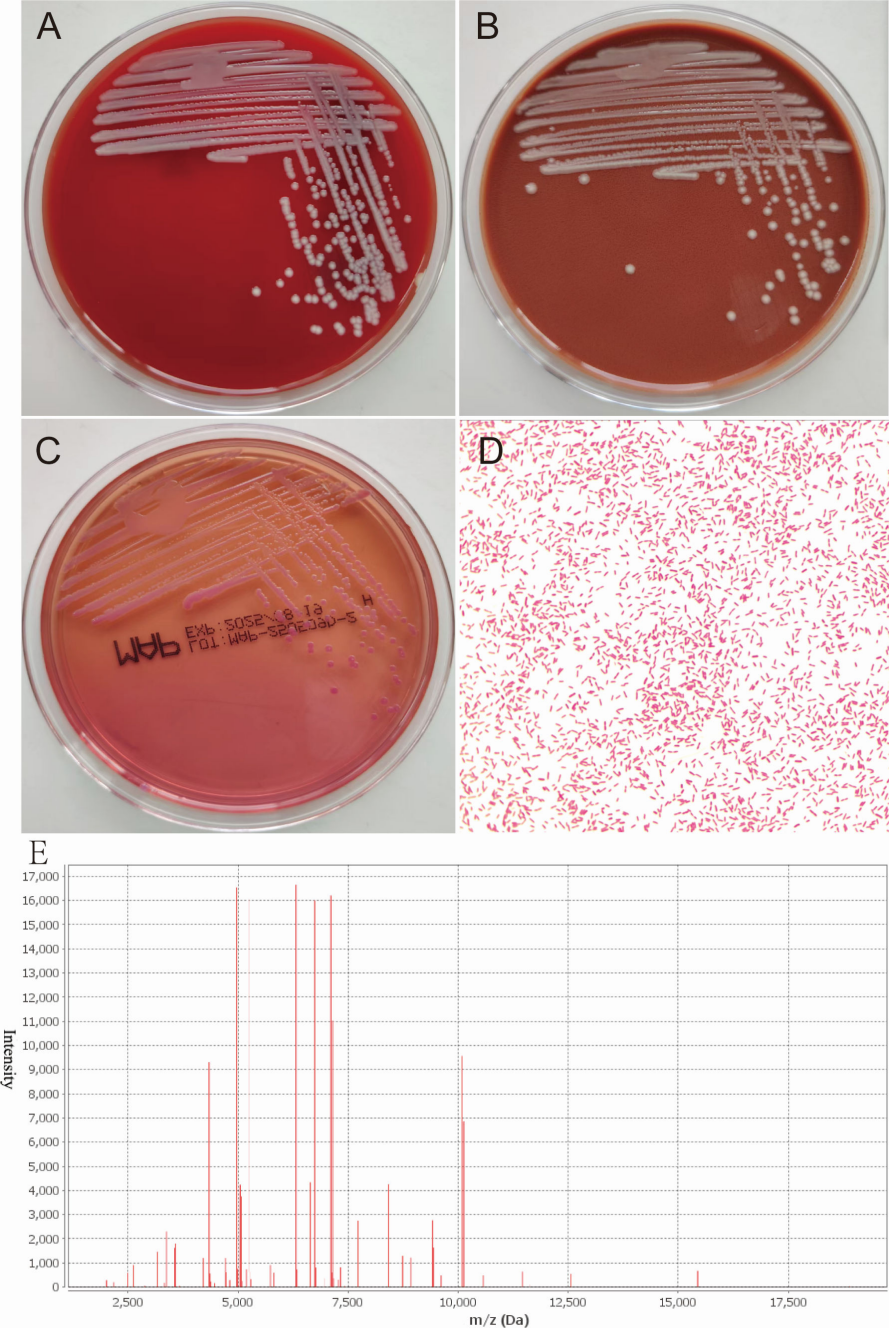
Colony morphology of *B. hinzii* on various agar plates and the results of mass spectrometry identification. Colony of strain on blood agar (**A**), chocolate agar (**B**), MacConkey agar (**C**), Gram staining of the colony (**D**), and MALDI-TOF MS analysis of the strain (**E**).

Three days later, the patient developed fever (37.9°C) and produced dark red, blood-tinged sputum. Laboratory investigations revealed leukocytosis (white blood cell count: 10.96 × 10⁹/L, normal reference range: 3.5–9.5 × 10⁹/L), neutrophilia (neutrophil count: 9.24 × 10⁹/L, normal reference range: 1.8–6.3 × 10⁹/L), and a significantly elevated high-sensitivity C-reactive protein (hs-CRP) level (119.13 mg/L, normal reference range: 0–6 mg/L). Chest CT scan demonstrated multiple pulmonary infiltrates, consolidation in both lower lobes, and a small amount of bilateral pleural effusion. These findings were consistent with the diagnosis of ventilator-associated pneumonia (VAP) as per the IDSA (Infectious Diseases Society of America) criteria. Representative CT images are shown in Fig. 1. Bronchoscopy was performed, and bronchoalveolar lavage fluid (BALF) was collected for microbiological analysis. After 48 h of incubation at 35°C with 5% CO₂, smooth, round, white colonies approximately 1 mm in diameter were observed on blood agar plates (Fig. 2A). Colonies grown on chocolate agar exhibited similar size and appearance to those on blood agar (Fig. 2B). On MacConkey agar, pink colonies approximately 1 mm in diameter were observed (Fig. 2C). The oxidase test was positive for the isolate. Gram staining and microscopic examination confirmed the presence of small Gram-negative rods (Fig. 2D). Identification using the VITEK MS system (Biomerieux, France), database version kb3.2, identified the isolate as *Bordetella hinzii (B. hinzii*), as shown in Fig. 2E. However, identification using the VITEK 2 system (Biomerieux, France) was unsuccessful. The identification was further confirmed by 16S rRNA gene sequencing performed at BGI Genomics, revealing 99.93% sequence homology to known *B. hinzii* strains in the NCBI database (data uploaded to NCBI GenBank: PV931814). The presence of *B. hinzii* was reconfirmed from an additional BALF specimen collected 4 days later.

Antimicrobial susceptibility testing was conducted using the GN13 card (Biomerieux, France) for Gram-negative bacteria on the VITEK 2 system. Results were interpreted based on the breakpoints for “Other Non-*Enterobacteriaceae*” as defined in CLSI M100, 33rd edition (1). The isolate demonstrated susceptibility to piperacillin/tazobactam, imipenem, amikacin, gentamicin, levofloxacin, and trimethoprim/sulfamethoxazole but showed resistance to ceftazidime, ceftriaxone, cefepime, and aztreonam (Table 1). At the time of pneumonia diagnosis, the clinician discontinued ceftriaxone/tazobactam and switched to piperacillin/sulbactam combined with levofloxacin for treatment. Levofloxacin was discontinued 5 days later, and piperacillin/sulbactam was discontinued 2 weeks later. Following treatment with susceptible antibiotics, the patient’s body temperature returned to normal and his symptoms improved significantly. By about 10 days after treatment initiation, a repeat sputum culture yielded negative results. He was subsequently transferred to a general ward for continued care and was successfully discharged five weeks after admission.

**TABLE 1:** Antimicrobial susceptibility results for *B. hinzii^a^*

Antibiotic	MIC (μg/mL)	Interpretation	S	I	R
Piperacillin/tazobactam	≤16/4	S	≤16/4	32/4–64/4	≥128/4
Ceftazidime	32	R	≤8	16	≥32
Ceftriaxone	≥64	R	≤8	16–32	≥64
Cefepime	≥64	R	≤8	16	≥32
Aztreonam	≥64	R	≤8	16	≥32
Imipenem	4	S	≤4	8	≥16
Amikacin	16	S	≤16	32	≥64
Gentamicin	2	S	≤4	8	≥16
Tobramycin	8	I	≤4	8	≥16
Ciprofloxacin	2	I	≤1	2	≥4
Levofloxacin	1	S	≤2	4	≥8
Trimethoprim/sulfamethoxazole	≤2/38	S	≤2/38	–	≥4/76

^*a*^ MIC, minimum inhibitory concentration; S, susceptible; I, intermediate; R, resistant.

## DISCUSSION

*B. hinzii* is a Gram-negative, small bacillus belonging to the genus *Bordetella* (family *Alcaligenaceae*), which comprises 16 species. It is primarily a respiratory pathogen in poultry, has been isolated from rodents, and rarely causes human infections—typically in immunocompromised individuals, often following exposure to animals carrying the organism (2, 3). A search using the keywords *B. hinzii* and human in PubMed and Web of Science revealed that infections caused by *B. hinzii* in immunocompetent individuals are rare. Most reported cases have occurred in immunocompromised patients, often following exposure to infected animals. In such patients, *B. hinzii* infections may lead to pulmonary infection, peripancreatic abscess, peritonitis, and brain abscess (4–7). Herein, we report a case of postoperative pulmonary infection caused by *B. hinzii* in an immunocompetent patient.

*B. hinzii*, involved in the current case, is clinically rare and was first isolated from human sputum in France in 1957, with formal nomenclature established in 1995 (8). *B. hinzii* primarily colonizes the respiratory tract of poultry, with droplet transmission being the main route of spread. Additionally, it may act as an opportunistic pathogen, causing respiratory infections in birds (9). In addition to poultry, it has also been reported to infect rabbits and mice (10). These animals may serve as potential reservoirs for transmission of the pathogen to humans. Fabre et al. reported that *B. hinzii* can persist in the human respiratory tract for over a year, indicating its ability to establish long-term colonization, in one case, mucoid colony morphology supported the hypothesis of prolonged carriage, similar to *Pseudomonas aeruginosa* in chronic infections. This persistent colonization complicates the identification of infection sources and suggests that *B. hinzii* may act as a latent opportunistic pathogen with delayed pathogenic potential (2). Previous case reports have shown that *B. hinzii* primarily causes pulmonary infections, often in patients with impaired immune function. In contrast to *Bordetella pertussis*, which typically induces paroxysmal coughing and a characteristic whooping sound (11), infections caused by *B. hinzii* usually do not present with such symptoms (12). In 2021, Chen et al. reported the first Asian case of *B. hinzii* infection, isolated from the sputum of a diabetic patient with pulmonary infection (13). After reviewing the existing literature, no cases of *B. hinzii* infections following pulmonary surgery have been documented. The patient in this case underwent pulmonary surgery due to trauma, had no diabetes history, and maintained normal immune function, highlighting that this pathogen can also infect immunocompetent patients. Thus, colonization of *B. hinzii* in the patient’s oropharynx, followed by pulmonary infection secondary to endotracheal intubation, is a likely route of transmission.

Fabre et al. reported that conventional automated biochemical identification systems are unable to accurately identify *B. hinzii* (2). Wang et al. described a case in which the VITEK 2 system misidentified *B. hinzii* as *Alcaligenes denitrificans subsp. xylosoxidans* (14). In the present case, the VITEK 2 system also failed to identify the organism, consistent with Fabre’s findings. Subsequent identification using the VITEK MS system yielded *B. hinzii* with a confidence level of 99.9%. This result was further confirmed by 16S rRNA gene sequencing, which showed 99.93% similarity to *B. hinzii*. These findings suggest that in laboratories lacking access to mass spectrometry, *B. hinzii* may go undetected or be misidentified as another species, and its true prevalence in human infections may, therefore, be underestimated.

Currently, there are no established interpretive criteria for antimicrobial susceptibility testing of *B. hinzii*, and standardized guidelines for antimicrobial selection are lacking. In the absence of defined EUCAST breakpoints for *B. hinzii*, Chahi and Collercandy assessed antimicrobial susceptibility based on pharmacokinetic/pharmacodynamic (PK-PD) parameters (15, 16). Chahi et al. reported a bloodstream infection in a SARS-CoV-2 infected patient in Belgium, in which the isolate was resistant to amoxicillin/clavulanic acid, ceftriaxone, and ciprofloxacin, but susceptible to piperacillin/tazobactam, ceftazidime, and meropenem; the patient’s symptoms improved significantly following treatment with piperacillin/tazobactam (15). Collercandy et al. described a case in France where *B. hinzii* was isolated from the urine of a patient with urinary tract infection. The strain was resistant to amoxicillin, cefotaxime, tobramycin, and ciprofloxacin, but susceptible to piperacillin/tazobactam, ceftazidime, carbapenems, gentamicin, and amikacin. Although no established breakpoint exists for trimethoprim/sulfamethoxazole, the MIC was low, and the patient had a favorable outcome with its use (16).

In this case, antimicrobial susceptibility testing of the *B. hinzii* isolate was performed using the GN13 card, following the methodology described by Maison-Fomotar et al. (17). Interpretive breakpoints were applied according to the “Other Non-*Enterobacteriaceae*” category in CLSI M100, 33rd edition. The isolate demonstrated high-level resistance to cephalosporins, including ceftriaxone, ceftazidime, and cefepime. Initial empirical treatment with ceftriaxone/tazobactam yielded no significant clinical improvement. The patient was subsequently switched to piperacillin/sulbactam and levofloxacin, resulting in marked symptomatic improvement.

In conclusion, this case demonstrates that *B. hinzii* can cause true postoperative pulmonary infections in immunocompetent patients. Accurate identification requires tools such as MALDI-TOF mass spectrometry and 16S rRNA sequencing, and treatment should be guided by susceptibility testing due to the lack of standardized breakpoints and variable resistance patterns.

## SELF-ASSESSMENT QUESTIONS

1. Which of the following is the most common reservoir for Bordetella hinzii?
  - Human nasopharynx
  - Poultry respiratory tract
  - Water
  - Domestic cats
2. Which laboratory method offers the highest accuracy for identifying Bordetella hinzii in clinical samples?
  - Conventional biochemical identification systems
  - MALDI-TOF mass spectrometry with updated databases
  - Gram stain alone
  - Oxidase and catalase tests
3. Which statement about Bordetella hinzii infection in humans is TRUE?
  - It only infects immunocompromised individuals.
  - It commonly causes pertussis-like symptoms with a whooping cough.
  - It can cause opportunistic infections.
  - It is primarily transmitted via contaminated water.

## ANSWER TO SELF-ASSESSMENT QUESTIONS

1. Which of the following is the most common reservoir for *Bordetella hinzii*?
  - Human nasopharynx
  - Poultry respiratory tract
  - Water
  - Domestic cats

Correct Answer: b. Poultry respiratory tract

*Bordetella hinzii* primarily colonizes the respiratory tract of poultry and may cause respiratory disease in birds. Humans are incidental hosts, and persistent colonization is rare in healthy individuals. The organism is not typically found in water, and there is no strong evidence to suggest domestic cats as a reservoir.

- Which laboratory method offers the highest accuracy for identifying *Bordetella hinzii* in clinical samples?
  - Conventional biochemical identification systems
  - MALDI-TOF mass spectrometry with updated databases
  - Gram stain alone
  - Oxidase and catalase tests

Correct Answer: b. MALDI-TOF mass spectrometry with updated databases

MALDI-TOF MS, when paired with an updated database, can reliably identify *Bordetella hinzii*. Conventional biochemical systems often fail to identify or misidentify the organism. Gram stain and basic enzymatic tests are useful for preliminary classification but are not species-specific.

- Which statement about *Bordetella hinzii* infection in humans is TRUE?
  - It only infects immunocompromised individuals.
  - It commonly causes pertussis-like symptoms with a whooping cough.
  - It can cause opportunistic infections.
  - It is primarily transmitted via contaminated water.

Correct Answer: c. It can cause opportunistic infections.

Human cases of *Bordetella hinzii* infection are rare but frequently occur after contact with animals carrying *Bordetella hinzii*. Unlike *Bordetella pertussis*, it does not typically cause paroxysmal coughing with a characteristic “whoop.” Transmission is mainly respiratory, not waterborne.

**TAKE-HOME POINTS**

- *Bordetella hinzii* is a rare opportunistic pathogen in humans, typically associated with poultry and other animal reservoirs, and can cause infection in both immunocompromised and immunocompetent individuals.
- Accurate identification is challenging; conventional biochemical systems may misidentify or fail to detect the organism, making MALDI-TOF MS and/or 16S rRNA sequencing the preferred diagnostic tools.
- Antimicrobial susceptibility patterns are variable, and standardized interpretive criteria are lacking; targeted therapy should be guided by *in vitro* susceptibility results.
- Awareness of *Bordetella hinzii* as a potential cause of postoperative or ventilator-associated pneumonia can facilitate timely diagnosis and effective management.
